# Peculiar Symptoms from Tape-Worm

**Published:** 1846-01

**Authors:** 


*Peculiar Symptoms from Tape-worm.*—Dr. Steinbeck observed in a woman 22 years of age a strange case of vertigo, with continual humming in both ears, for which she could assign no cause whatever. The complaint had already lasted a year, and many internal and external remedies had been employed, but with no benefit. The humming appeared first, increased gradually in strength, and was at last accompanied with the giddiness before mentioned, which occurred from two to four times a day. Congestion of blood to the head being the supposed cause, leeches, foot-baths, and purgatives were employed. After the patient had been treated inefficiently by the above remedies for four weeks, the author was called in, and considered the disorder to proceed from abdominal nervous derangement. He ordered, therefore, antihysterics, but to no purpose. All on a sudden violent vomiting ensued, which yielded at length to prussic acid. The patient took two drops every two hours, when the vomiting ceased after a few doses, and the patient felt a certain ease, which she had not perceived for two years. The nervous abdominal symptoms—retching, humming, and giddiness—had entirely vanished. The cause of these complaints soon became evident, for on the following day a perfect, dead tape-worm, fifteen yards long, was evacuated, though the want of the usual helminthic symptoms neither suggested to the first physicians nor to the author the proper diagnosis. The strong doses of prussic acid had undoubtedly killed the worm. Since the latter has been evacuated, the woman is perfectly healthy, and no longer suffers from the previous giddiness or humming.—*Ranking's Abstract* from *Pr. Vereins-zeitung*,—*Med. News.*

